# Microwave-assisted one-pot synthesis of CrMnFeCoNi multimetallic nanoparticle-loaded UiO-66 for visible-light photocatalytic degradation of methylene blue

**DOI:** 10.1039/d6ra03021a

**Published:** 2026-05-18

**Authors:** Mostafa Khajeh, Mansour Ghaffari-Moghaddam, Afsaneh Barkhordar

**Affiliations:** a Department of Chemistry, Faculty of Science, University of Zabol Zabol Iran m_khajeh@uoz.ac.ir; b Advanced Materials & Manufacturing Laboratory, University of Zabol Zabol Iran

## Abstract

The increasing contamination of water resources with synthetic dyes, particularly methylene blue (MB), from textile industries necessitates the development of efficient and environmentally friendly remediation technologies. Herein, the first-time synthesis of multimetallic nanoparticles with an equimolar ratio of Fe, Co, Cr, Mn, and Ni, anchored on UiO-66 metal–organic framework (MM@UiO-66), is presented using microwave-assisted one-pot synthesis. The nanocomposite was well characterized using powder X-ray diffraction (PXRD), Fourier-transform infrared spectroscopy (FT-IR), scanning electron microscopy (SEM), and thermogravimetric analysis (TGA), thereby confirming retention of the UiO-66 framework and incorporation of multimetallic species in near-equimolar proportions. Photocatalytic performance was evaluated *via* visible-light-driven degradation of MB under optimal conditions. The MM@UiO-66 composite showed excellent photocatal e, achieving 95.1% MB degradation under low-power LED light illumination. Parametric systematic research under optimized conditions revealed the optimal performance at 20 mg catalyst loading, pH 7.5, and 10 mg L^−1^ initial MB concentration. Kinetic analysis showed that MB was degraded rapidly, following a pseudo-first-order reaction model. Owing to its stable framework, MM@UiO-66 maintained strong catalytic performance and could be reused efficiently through six successive cycles without significant loss of activity. Scavenger experiments confirmed hydroxyl radicals (˙OH) and photogenerated holes (h^+^) as predominant oxidative species. The synergistic combination of UiO-66's chemical tunability and high surface area and the multi-element active sites of the multimetallic nanoparticles led to enhanced photocatalysis compared to conventional systems, indicating excellent potential for sustainable treatment of wastewater.

## Abstract 2

The increasing contamination of water resources with synthetic dyes, particularly methylene blue (MB), from textile industries necessitates the development of efficient and environmentally friendly remediation technologies.

## Graphical abstract

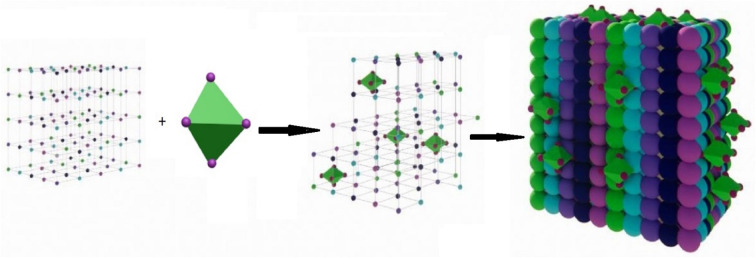

## 1. Introduction

Water is a resource essential to human health and the environment.^1^ However, the supply of safe drinking water is coming under growing stress owing to rapidly developing populations, industrialization, climate change, and rising pollution.^1^ Amongst the pollutants threatening water, artificial dyes released from the textile, leather, and printing industries have been of particular concern in light of their toxicity, persistence, and resistance to conventional treatment methods.^2,3^ MB is a popular cationic phenothiazine dye with extensive application as a textile dye, leather dye, and printing dye, and as a bio-stain in biochemical assays. It is a prototypical example of these challenges.^4,5^ Though it has beneficial applications, long-term use of MB in concentrations above 5 mg L^−1^ may cause skin irritation, methemoglobinemia, and DNA damage in mammalian cells,^6^ and its intense color reduces water transparency by more than 90% at 10 mg L^−1^, hindering aquatic photosynthesis.^7^ Additionally, MB possesses a long half-life in the environment (460 days) with bioaccumulation potential, threatening aquatic organisms such as Daphnia magna even at sublethal dosages (0.8 mg L^−1^).^8^ Hence, the search for effective approaches to degrade MB in wastewater has become a pressing issue in environmental protection. Traditional physical, chemical, and biological treatment techniques often fall short.^9–11^ However, due to the intrinsic limitations of most of today's photocatalytic systems—*i.e.*, insufficient active sites, unstable performance, and poor electron transfer efficiency-the demand to create more efficient catalysts is still present. In this contribution, we synthesized an MM supported on UiO-66 to create a nanocomposite with abundant active sites, excellent stability, and excellent light-driven catalytic activity. This study demonstrates outstanding performance toward dye degradation, particularly methylene blue, and has great potential for eco-friendly wastewater treatment.

Metal–organic frameworks (MOFs) have proven to be a convenient family of porous materials characterized by high surface areas, porous structures that can be tailored, and the ability to incorporate diverse functional groups through relatively simple synthetic pathways.^12,13^ MOFs are valuable candidates for the removal of pollutant from water due to their built-in porosity and chemical tunability, which complement tenets of green materials.^14^ Among them, zirconium-based MOFs such as UiO-66 have attracted special attention due to their excellent chemical stability, aqueous stability, and fine solubility, all of which enhance their performance and bioavailability in environmental processes.^15^ Some studies have established the excellent capability of Zr-MOFs to adsorb and remove a range of pollutants, especially colored pollutants, from water systems.^16^ Their water resistance enables them to be structurally stable and retain adsorption capacity under extended working conditions during wastewater treatment processes.^17^ Compared to conventional adsorbents, MOFs have a better structural diversity, the ability to change pore size and surface chemistry through metal node and organic linker modification, and comparatively minimal regeneration energy requirements combined with good thermal and mechanical stability.^18^ Yet, certain constraints prevent their broader application. Specifically, water can destroy coordination bonds within the frameworks, resulting in hydrolysis and a decrease in adsorption or catalytic performance.^19^ Second, although UiO-66 is reasonably stable, incorporating bulky organic units has the potential to decrease its porosity and surface area—key characteristics for efficient adsorption and catalysis.^20^ Other limitations of most MOFs are limited electrical conductivity and poorer electrocatalytic activity.^21^

High-entropy alloy nanoparticles (HEANPs) are composed of five or more metal elements in near equimolar ratios.^22,23^ The unique multicomponent composition of HEANPs provides a high density of free electrons, which enables their electrocatalytic activity and general electrochemical performance, making them outstanding catalysts. The HEAs allow for charge separation in photocatalytic systems due to their multi-element nature and lattice disordering. Compositional heterogeneity and structural distortions generate defect sites and internal electric fields that quench the recombination of electrons and holes. Localized charge redistribution caused by differences in the electronic structures of constituent elements results in directional carrier migration. The inherent metallic conductivity of HEAs allows for efficient electron transport to reaction sites. Such synergistic effects enhance carrier lifetime and overall photocatalytic efficiency.^24–27^ Notably, the five-element equimolar MM composition supported on UiO-66 has not been previously reported for photocatalytic applications, and the microwave-assisted one-pot synthesis approach, which simultaneously builds the MOF structure and encapsulates MM nanoparticles, represents a significant advance over conventional multi-step processes.

**Scheme 1:**
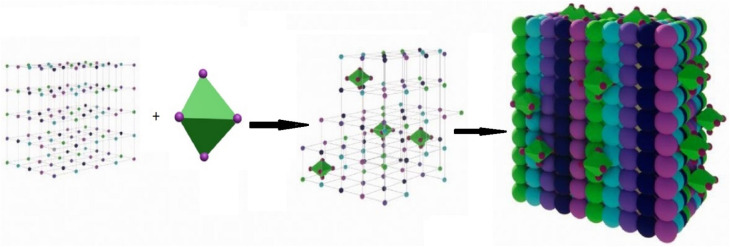
Illustration of the process of MM@UiO-66.

In the present study, we report the synthesis of MM multimetallic nanoparticles anchored on a UiO-66 metal–organic framework *via* a rapid microwave-assisted one-pot approach (Scheme 1). The primary objective of this work is to develop an efficient visible-light-driven photocatalyst for methylene blue degradation, while simultaneously introducing a simplified and time-efficient synthesis strategy. The structural and physicochemical properties of the resulting composite were comprehensively characterized, and the key parameters governing photocatalytic performance were systematically evaluated. Despite significant progress in photocatalytic materials, many existing systems remain limited by their reliance on ultraviolet irradiation, use of chemical additives, complex multi-step synthesis routes, and inadequate stability during reuse. In contrast, the MM@UiO-66 composite developed in this work leverages the synergistic interaction between multi-element active sites and a porous framework to enable efficient, additive-free photocatalysis under visible light. This integrated design, combined with a rapid one-pot synthesis method and demonstrated reusability, highlights the potential of this system as a practically viable and scalable approach for wastewater treatment.

## 2. Experimental

### 2.1. Chemicals

All received chemical compounds from suppliers were used. Reagent grade highest purity available zirconium(iv) chloride (ZrCl_4_), chromium(iii) chloride hexahydrate (CrCl_3_·6H_2_O), ferric chloride hexahydrate (FeCl_3_·6H_2_O), cobalt(ii) chloride hexahydrate (CoCl_2_·6H_2_O), manganese(ii) chloride tetrahydrate (MnCl_2_·4H_2_O), nickel(ii) nitrate hexahydrate (Ni(NO_3_)_2_·6H_2_O), (all supplied by Merck, Darmstadt, Germany) were used. Terephthalic acid, methanol, acetonitrile, ethanol, and *N*,*N*-dimethylformamide (DMF) were obtained from Sigma-Aldrich, a commercial source.

### 2.2. Instruments

Powder X-ray diffraction (PXRD) patterns were recorded on a Philips Xpert diffractometer (Amsterdam, The Netherlands) using monochromatized Cu Kα radiation (*λ* = 1.5418 Å) between the scanning range of 2*θ* = 1.5–80°. Fourier transform infrared (FT-IR) spectra were recorded in the range of 460–4000 cm^−1^ by using KBr pellets on a PerkinElmer Spectrum FT-IR spectrometer (version 10.01.00, USA). The surface morphology of the samples was investigated by scanning electron microscopy (SEM; MIRA3 TESCAN, Czech Republic). UV measured the analyte concentration in the solution UV-vis spectrophotometry (UV-2100 RAY, Leigh, Beijing, China) at the absorption maximum wavelength (664 nm). The pH values of the solutions were determined using a Metrohm 630 pH meter with a glass electrode. Thermogravimetry analysis (TGA) was carried out on a Mettler Toledo TGA/DSC instrument under the conditions of a nitrogen environment.

### 2.3. Synthesis of MM@UiO-66

For the synthesis of the MM@UiO-66 nanomaterials, microwave-assisted heating was employed. First, a metal solution was prepared by dissolving 11.65 mg of zirconium chloride (ZrCl_4_, 0.05 mmol) in 4 mL of DMF. To this solution, five equimolar metal salts of the elements were added and stirred (all 0.01 mmol, *i.e.*, a total of 0.05 mmol). Next, 16.6 mg of the organic ligand terephthalic acid (H_2_BDC, 0.1 mmol) and 0.5 mL of acetic acid, serving as a modulator, were added to the solution, which was stirred magnetically at 60 °C for 10 minutes until a homogeneous solution was achieved. The resultant volume was then made up to 10 mL with DMF. The solution so obtained was transferred to a microwave reaction vessel. Microwave conditions were also optimized at 120 °C and 300 W for 20 min. After the reaction was completed, the system was allowed to cool to room temperature under atmospheric conditions, and the resulting solid product was recovered by centrifugation. The precipitate was washed twice with DMF and twice with ethanol to remove impurities. Finally, the sample was dried in an oven for 16 h at 60 °C.

### 2.4. Photodegradation measurement

The photocatalyst activity of the MM@UiO-66 nanoparticles was explored through the degradation of the pesticide methylene blue. For a typical photocatalytic test, 20 mg of the MM@UiO-66 photocatalyst was dispersed in 25 mL of an aqueous methylene blue solution with an appropriate concentration and stirred in the dark for 10 min to attain adsorption–desorption equilibrium. Before visible-light irradiation, the suspension was stirred in the dark for 10 min to establish adsorption–desorption equilibrium. Preliminary tests displayed that extending the dark adsorption period beyond 10 min did not produce a significant additional decrease in MB concentration, confirming the adequacy of the selected equilibration time. Suspension was then exposed to visible-light irradiation from 280 LED lamps (1 W, 3.2 V) arranged in a circular configuration, resulting in an intensity of 32 000 lux. Cooling fans maintained the reaction temperature at 28–34 °C in a Heber photoreactor. The LED bulbs operate in the visible spectrum, with the maximum emission wavelength between 400 nm and 700 nm, with the most intense emissions occurring at 460 nm (blue). The illumination intensity is 85 mW cm^−2^, as measured by a calibrated radiometer on the walls of the reactor chamber. Aliquots of 2 mL were withdrawn at periodic intervals under illumination and centrifuged to precipitate the catalyst particles. UV-vis analysis was performed on the resulting clear supernatant by a UV-vis spectrophotometer, monitoring the characteristic absorption peak of methylene blue at 664 nm. The photocatalytic degradation performance of the MM@UiO-66 nanocomposite was then calculated using the following equation:

1 Degradation efficiency(%) = [1 − *C*_*t*_/*C*_0_] × 100

where *C*_0_ is the initial concentration of the methylene blue solution and *C*_*t*_ is the concentration of methylene blue at different time intervals.

## 3. Results and discussion

### 3.1. Characterization

**Fig. 1:**
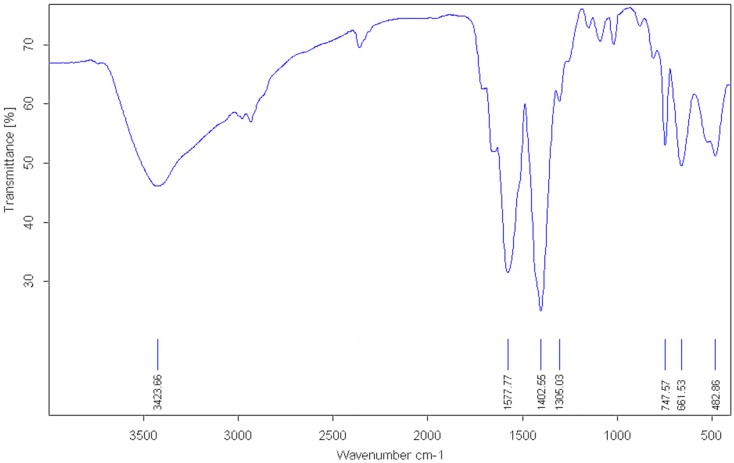
FT-IR spectrum of MM@UiO-66.

The nanocomposite's functional groups and chemical structure were examined by Fourier-transform infrared (FTIR) spectroscopy between 400 and 4000 cm^−1^ (Fig. 1). The FTIR spectrum contains characteristic bands for the UiO-66 metal–organic framework structure. The widened absorption band at around 3432 cm^−1^ represents the O–H stretching vibration of hydroxyl groups within the Zr_6_O_4_(OH)_4_ clusters as well as adsorbed water molecules within the UiO-66 framework. The strong bands at 1577 cm^−1^ and 1402 cm^−1^ can be assigned to the asymmetric and symmetric stretching vibrations of the carboxylate groups (COO^−^) attached to the Zr_6_O_4_(OH)_4_ clusters, respectively, which confirm effective coordination of Zr–O–C bonds within the UiO-66 structure. The characteristic peaks of typical absorption occurring in the fingerprint region at 747, 661, and 482 cm^−1^ are attributed to the out-of-plane bending of benzene rings of C–H, stretching vibrations of Zr–O, and vibrational modes of the Zr-oxo clusters, respectively. The peaks are consistent with previously reported FTIR spectra for pure UiO-66 materials, which confirm that the framework structure is not affected upon nanocomposite incorporation. The absence of additional absorption bands attributable to the MM phase in the FTIR spectrum is consistent with the relatively low MM content in the composite and the overlapping of any weak metal-related vibrations with the dominant UiO-66 framework bands. The successful incorporation of MM nanoparticles is instead confirmed by PXRD, SEM, and ICP-OES analysis, as discussed below. Therefore, the presence of MM nanoparticles is indirectly confirmed by the preservation of all characteristic UiO-66 absorption bands without causing extensive structural devastation. The continuous intensity and position of the framework vibrational modes ensure that the MM nanoparticles have been successfully deposited on the UiO-66 surface by physical anchoring and not by chemical coordination, with the organic–inorganic framework integrity of the composite material preserved.

**Fig. 2:**
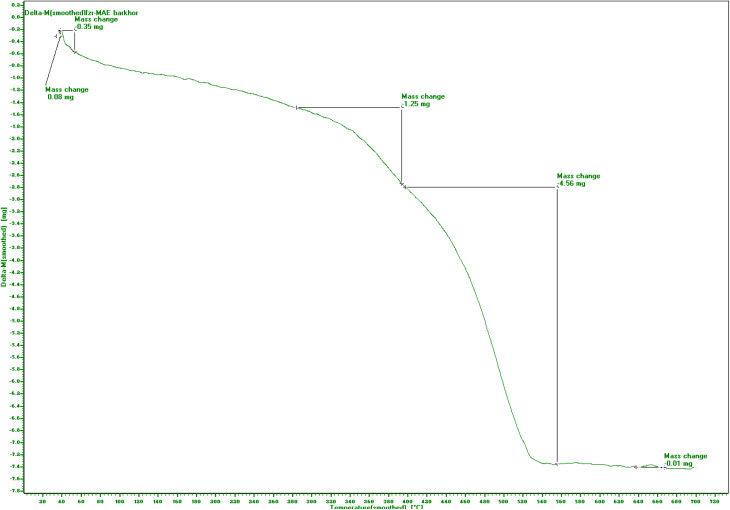
TG profile of MM@UiO-66.

Thermogravimetric analysis of the MM@UiO-66 composite (Fig. 2) exhibits three distinct areas of mass loss. The first mass loss, below *T*_1_ = 100 °C and amounting to Δ*m*_1_ = 0.35 mg, is caused by desorption of physisorbed water and excess solvent out of the pores of the UiO-66. A second, minor mass loss between *T*_1_ and *T*_2_ = 420 °C (Δ*m*_2_ = 1.25 mg) likely reflects desorption of solvent molecules coordinated to the Zr_6_ clusters (*e.g.*, DMF or residual synthesis species) and partial dihydroxylation. The principal decomposition process occurs between *T*_2_ and *T*_3_ = 560 °C, accompanied by a mass loss of Δ*m*_3_ = 4.56 mg and is attributed to the thermal degradation of terephthalate linkers, resulting in the subsequent collapse of the UiO-66 pore structure. At higher temperatures above *T*_3_, the sample attains a residual mass plateau, attributable to decomposition of the Zr-based nodes into ZrO_2_ and the metal content (metal or metal-oxide residues). These properties are consistent with reported thermal properties of UiO-66 and confirm that the structure remains stable until *T*_2_ before ligand degradation.^28^

**Fig. 3:**
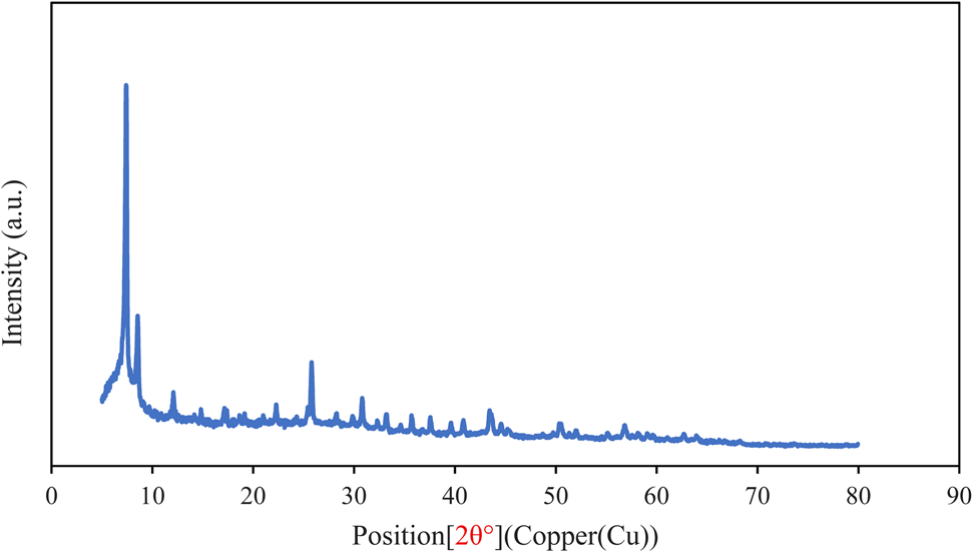
PXRD pattern of MM@UiO-66.

PXRD patterns of MM@UiO-66 are shown in Fig. 3. Characteristic diffraction peaks of UiO-66 are observed at 2*θ* = 7.43°, 8.58°, 12.09°, 17.14°, and 25.78°, confirming preservation of the crystalline UiO-66 framework after synthesis. Additional reflections are observed in the 43–51° region; however, due to overlap with UiO-66 reflections and the relatively low loading of multimetallic species within the dominant UiO-66 matrix, these peaks cannot be unambiguously assigned to a distinct metallic phase. Reflections observed between 60° and 73° are attributed to higher-order UiO-66 diffraction peaks. Therefore, PXRD analysis in this study is used primarily to confirm retention of the UiO-66 structure after synthesis, while successful incorporation of MM multimetallic species is supported by ICP-OES analysis and TEM observations.^29,30^

**Fig. 4:**
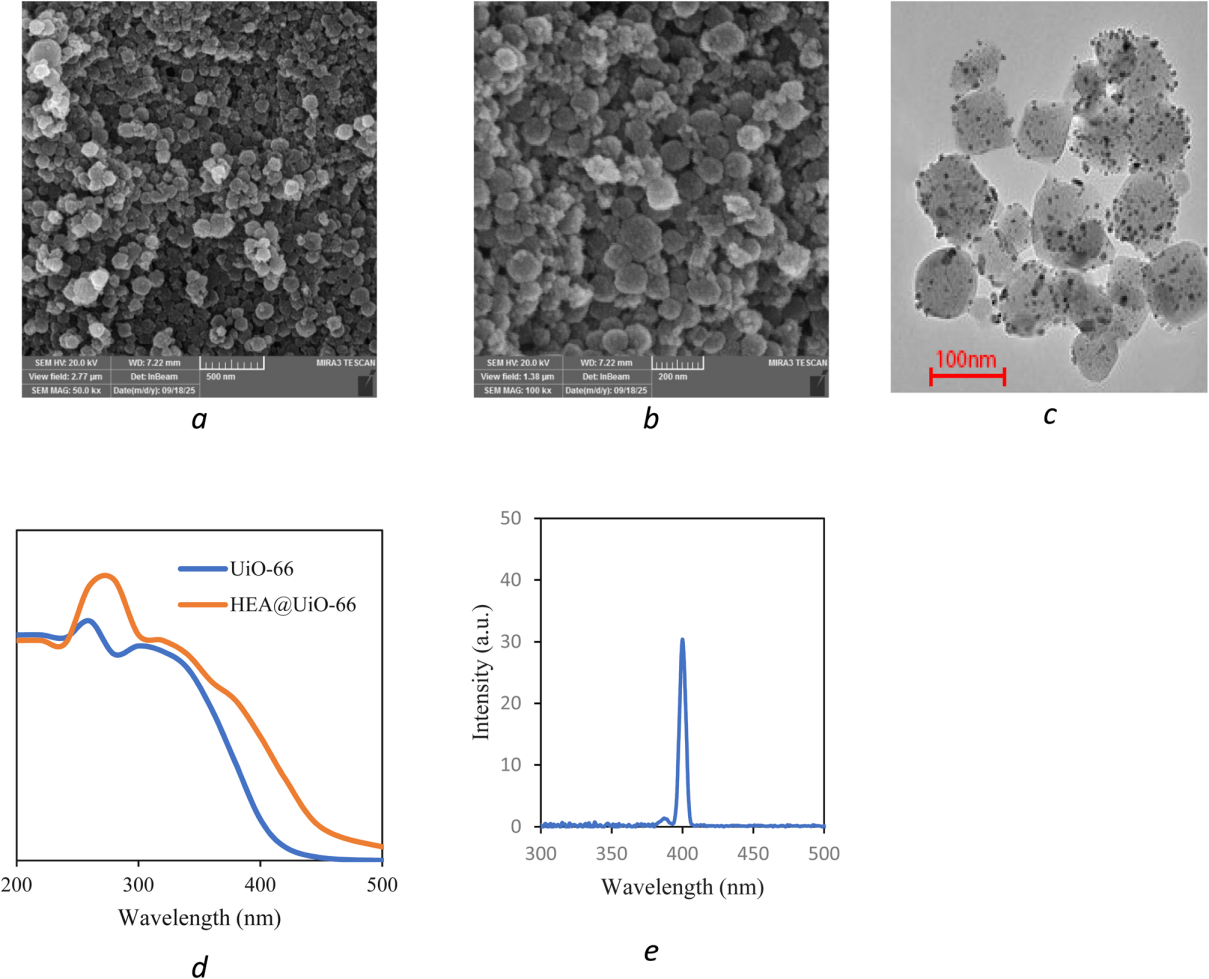
The SEM images (a and b), TEM (c), UV-vis diffuse reflectance spectra (d), and photoluminescence emission spectrum (e) of MM@UiO-66.

The SEM images of the MM@UiO-66 nanocomposites are shown in Fig. 4a and b, indicating particles with a rounded morphology and an apparently rough surface texture. The TEM image of the MM@UiO-66 nanocomposite is shown in Fig. 4c, where nanoscale dark-contrast features are observed on the surfaces of the UiO-66 particles. These higher electron-density regions are consistent with the presence of incorporated multimetallic species relative to the MOF matrix. The simultaneous presence of Cr, Mn, Fe, Co, and Ni was further verified by ICP-OES compositional analysis. The UV-vis diffuse reflectance spectra of UiO-66 and MM@UiO-66 are displayed in Fig. 4d. The two compounds display typical peaks at around 250 and 300 nm corresponding to ligand-to-metal charge transfer transitions from the Zr–O cluster and π–π* transitions in the benzene dicarboxylate linker, respectively. Importantly, MM@UiO-66 shows strong absorption peaks in the near-infrared region (>350 nm), indicating successful integration of the element into the UiO-66 MOF structure. The photoluminescence emission spectrum of the MM@UiO-66 nanocomposite is shown in Fig. 4e, where it can be seen that there is a clear, sharp, and intense peak centered around 400 nm. The emission peak is due to the radiative recombination of photo-induced charges inside the UiO-66 structure, which is similar to the ligand fluorescence described in earlier studies on Zr-MOFs. PL emission suggests altered charge recombination behavior after metal incorporation.

To provide quantitative proof of the MM makeup of the element phase in the composite material, ICP-OES testing was carried out on acid-dissolved MM@UiO-66 samples. The findings indicated the presence of chromium, manganese, iron, cobalt, and nickel in amounts of 0.95%, 0.98%, 1.15%, 0.94%, and 1.24% wt% by weight, respectively, representing approximately equal molar concentrations of all five metal elements. The predominant zirconium concentration at 94.7 wt% can be attributed to the bulk UiO-66 matrix in the composite, as expected from the compositional makeup of the sample.

### 3.2. Photocatalytic degradation of MB over MM@UiO-66 photocatalyst

#### 3.2.1. Factors affecting the activity

**Fig. 5:**
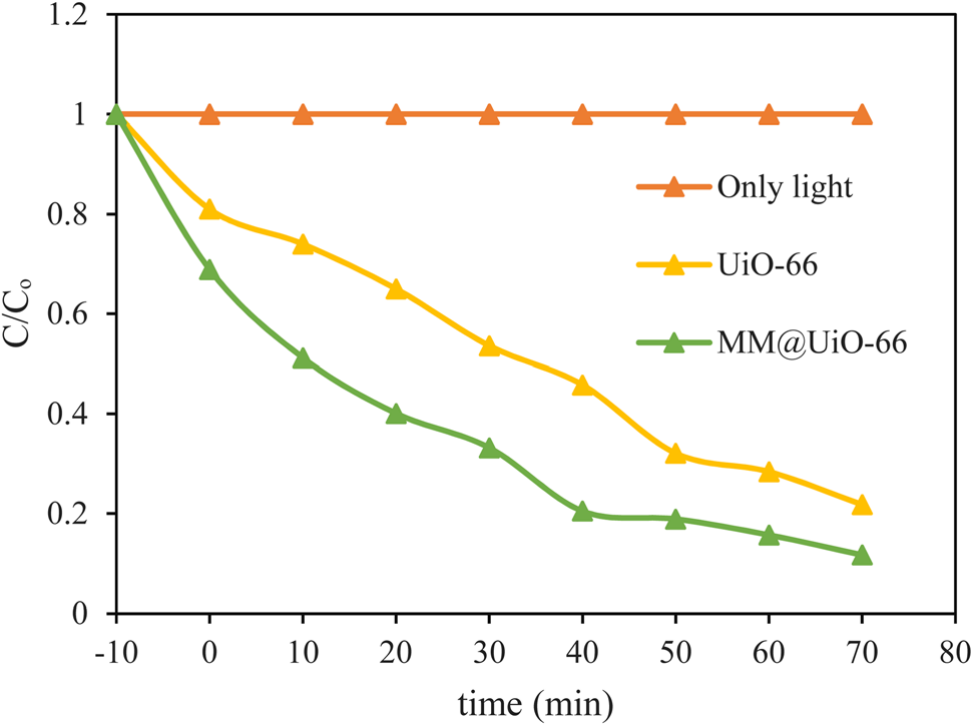
Degradation of MB using UiO-66 and MM@UiO-66 (10 mg L^−1^; 20 mg).

The photocatalytic performance of the photocatalysts (UiO-66 and MM@UiO-66 NPs) was assessed through monitoring the degradation of MB solutions upon visible light irradiation. As indicated by Fig. 5, no observable change is detected in the photocatalytic degradation experiment of the MB in the absence of any catalyst after 70 min of irradiation, thereby guaranteeing that MB is photochemically stable under the conditions employed. Initially, adsorption–desorption equilibrium occurred between the samples and the photocatalyst after incubation for 10 min in darkness. Pristine UiO-66 exhibited a gradual decrease in *C*/*C*_0_, indicating a moderate photocatalytic activity because of its Zr–oxo clusters and porous structure, which can adsorb and degrade MB to a certain degree. Notably, MM@UiO-66 exhibited a significantly faster and more pronounced decrease in MB concentration, achieving approximately 80–90% degradation in 70 minutes, compared with approximately 75–80% for UiO-66 and no impact for light alone. This enhanced activity arises from the synergy between the MM phase and the UiO-66 support: the transition-metal centers provide abundant active sites and broaden the visible-light absorption spectrum, while UiO-66 maintains a high surface area, thereby suppressing recombination of the photogenerated charge carriers.

**Fig. 6:**
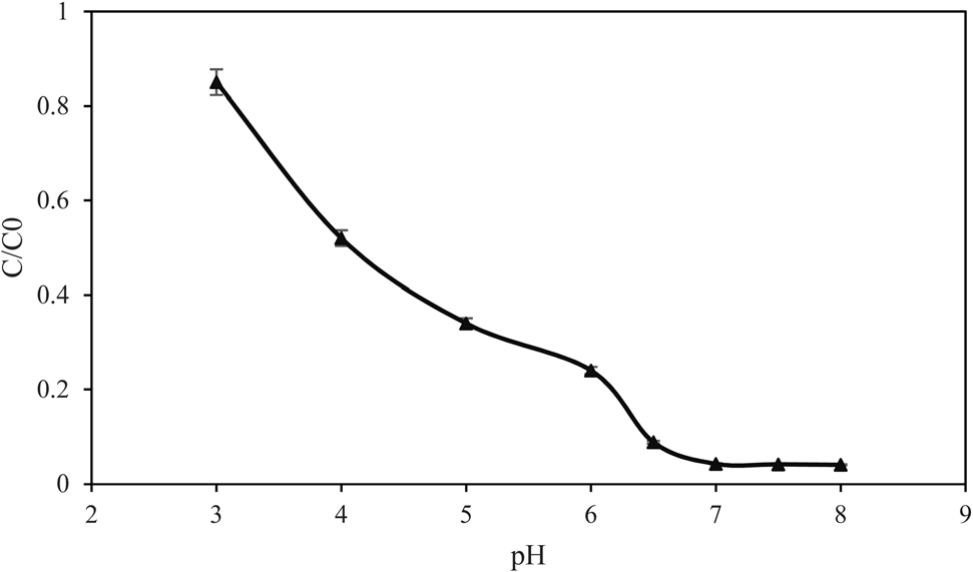
pH effect on the degradation of MB. Experimental conditions: concentration of MB, 10.0 mg L^−1^, amount of photocatalyst, 20 mg.

The photocatalytic effectiveness is usually multi-dimensional. The factors that matter most are the initial pollutant concentration, the dose of the catalyst, and the pH of the solution. Therefore, these three factors were investigated to find out if they have a bearing on the degradation of the dye. Fig. 6 indicates *C*/*C*_0_ of MB following 10.0 min dark equilibration and 60.0 min irradiation *versus* pH. The MM@UiO-66 composite exhibits strong pH-dependent degradation efficiency. The *C*/*C*_0_ is high at pH 3 but steadily decreases with increasing pH to values <0.05 at pH 6.5–8. The abrupt transition is the same as the established isoelectric point of the composite (pH 6.2). Below the isoelectric point, the composite surface becomes positively charged due to protonation of the surface groups, and, additionally, MB is a cationic (positive) dye. This results in electrostatic repulsion with the cationic MB dye and unfavourable adsorption. Above pH 6.2, the composite surface becomes negative, which enables strong adsorption of MB on active sites. The dark-stage adsorption occurs before light accumulates MB at the surface, making the photocatalytic degradation more effective under light. More OH^−^ is available at higher pH, hence increasing hydroxyl radical generation and redox potential change, further enhancing photocatalytic degradation. The combined effect of surface charge and reactive oxygen species explains the dramatic rise in degradation efficiency at pH greater than 6.2. The results confirm that the composite works best at slightly basic to neutral pH and that pH optimization can control its activity for cationic dye degradation.

**Fig. 7:**
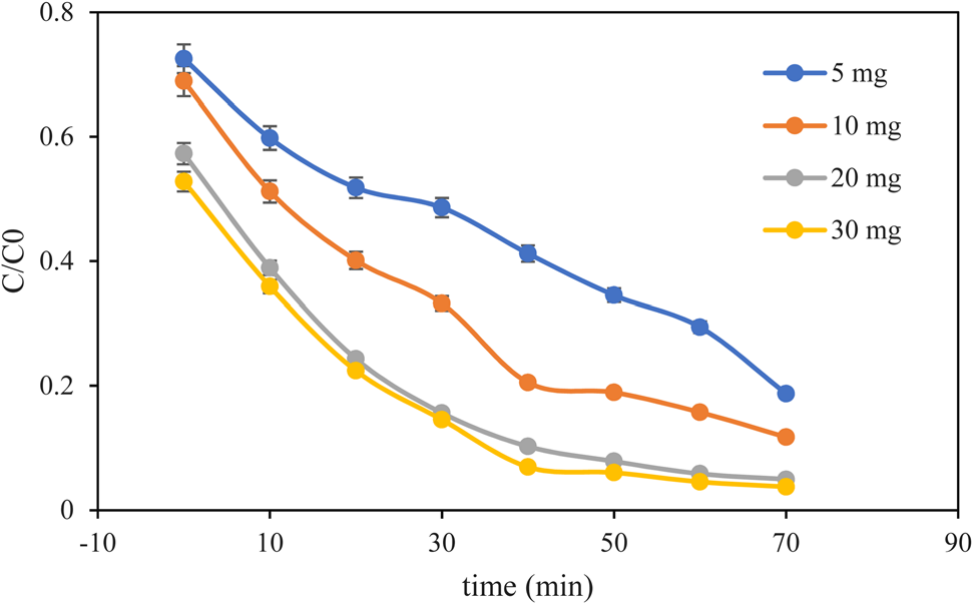
Photocatalyst dosage effect on the degradation of MB. Experimental conditions: concentration of MB, 10.0 mg L^−1^, pH 7.5.

We observed that the increase in the photocatalyst dose enhances the MB degradation (Fig. 7). Herein, several doses of photocatalyst were investigated on the degradation of MB. The results showed that the lowest amount of degradation was exhibited by 5.0 mg of the catalyst. In addition, the reaction rate increased with the increase in the dose of the photocatalyst, as indicated in Fig. 7. This is because the increase in the number of catalyst molecules enhances the surface-active sites and hence the efficiency at higher doses. Moreover, the intensity of reactive oxygen species formation also enhances with the increased dose of the catalyst. However, degradation efficiency becomes saturated at higher doses of the catalyst. So, 20 mg of photocatalyst was used for the next experiments.

**Fig. 8:**
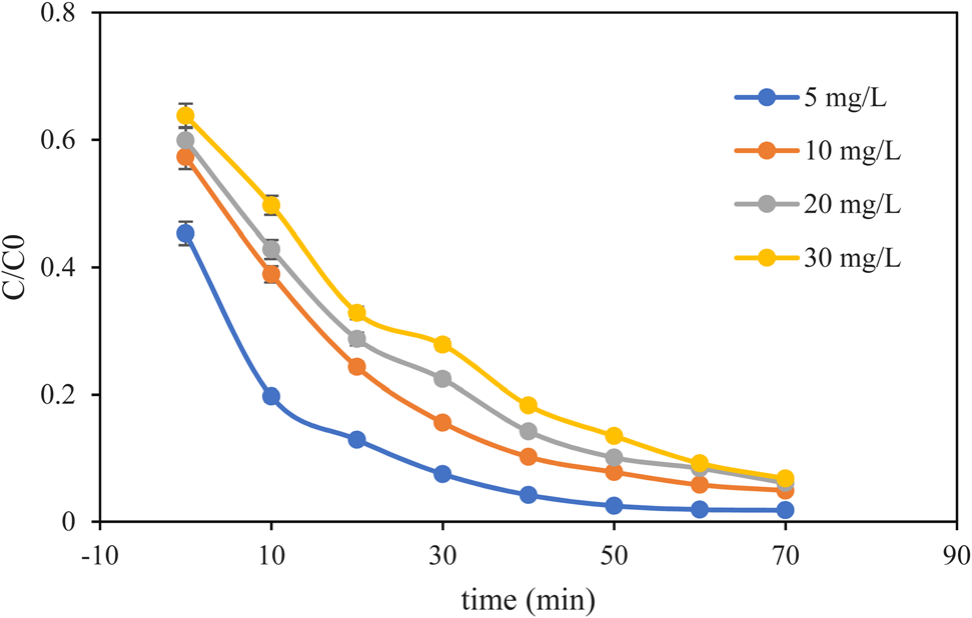
Concentration of MB effect on the degradation of MB. Experimental conditions: pH 7.5, catalyst dosage 20 mg.

The impact of the initial MB concentration on the photocatalytic efficiency of MM@UiO-66 degradation was studied under visible-light irradiation, and the data are shown in Fig. 8. For all the concentrations studied, the efficiency of MB degradation showed a typical rise with irradiation time, implying effective catalytic degradation. However, the degradation rate decreased as the initial MB concentration increased from 5 mg L^−1^ to 30 mg L^−1^. At the lowest concentration (5 mg L^−1^), almost complete removal of MB was achieved within 40 min, and at 30 mg L^−1^, >81% removal was achieved within the same time. The reason for such a trend is the smaller number of available active sites on the surface of the photocatalyst than the number of MB molecules at higher concentrations, which results in competitive adsorption and reduced degradation efficiency. Additionally, since the concentration of dye is high, the colored solution is more, and it is possible to decrease light penetration and reactive species formation. Better activity at low MB concentration is a reflection of the high intrinsic activity of the MM@UiO-66 composite material, while the gradual decrease in efficiency with growing concentrations is a typical mass transfer and light blocking limitation in heterogeneous photocatalysis. These results confirm that MM@UiO-66 exhibits efficient photocatalysis over a wide range of MB concentrations, with optimum efficiency at lower initial dye loadings.

#### 3.2.2. Reusability of MM@UiO-66

**Fig. 9:**
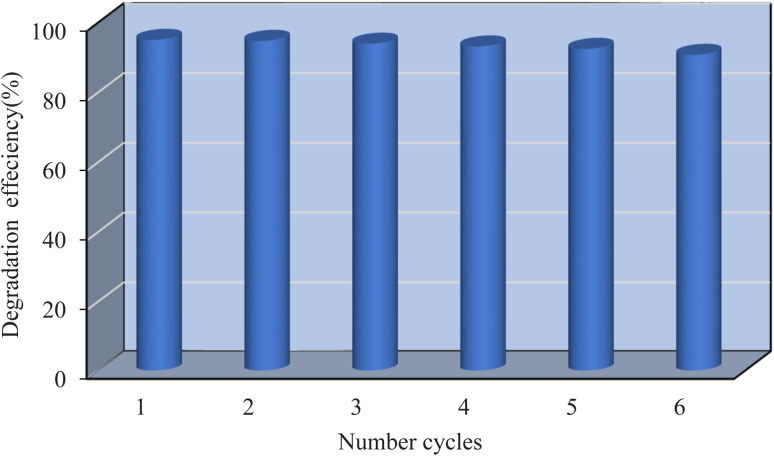
The reusability of MB in the optimized conditions.

Six successive adsorption–desorption cycles were performed to test the long-term stability and recyclability of the adsorbent material. 20 mg of the MM@UiO-66 composite was added to 20 mL of methylene blue (MB) solution of initial concentration 10 mg L^−1^ in each cycle, and was agitated for 30 min. After every run, the catalyst was washed with acetonitrile, then washed with distilled water, dried at 100 °C, and reused in the subsequent cycle. During these experiments, the MM@UiO-66 maintained the majority of degradation efficiency, with efficiency diminishing only marginally from 95.1% to 90.8% after six applications. Such performance indicates good chemical bonding between the metallic sites and organic linkers, which confers high structural stability and loss of activity. Because MB molecules have a loose association with the surface of the adsorbent, they are easily desorbed into the solution upon regeneration while the material remains intact.^12^ Such behavior indicates preservation of crystallinity and chemical integrity of the MM@UiO-66 hybrid upon repeated removal of MB. Though traces of MB remain even after washing. The outcome of these reusability tests is shown in Fig. 9.

#### 3.2.3. Total organic carbon (TOC) content

Methylene blue mineralization performance over the MM@UiO-66 catalyst was further evaluated by means of TOC analysis. As shown in Fig. S1, normalized TOC (TOC/TOC_0_) reduced successively with reaction time, which indicates successive degradation and mineralization of MB. During the initial 20 min, a TOC removal of 20–25% was observed, indicating fast degradation of dye molecules because of effective adsorption and catalytic oxidation on the MM@UiO-66 surface. A gradual reduction of TOC was noted after 20 min, with ∼90% mineralization being achieved after 70 min of reaction. This result not only confirms that the catalytic system bleaches MB but also that it effectively degrades organic intermediates to lower molecules and ultimately CO_2_ and H_2_O. The successive drop in TOC also shows that the degradation process undergoes sequential oxidative steps whereby aromatic intermediates are progressively oxidized to mineralization. This improved performance of MM@UiO-66 is attributed to some synergistic effects. The MM nanoparticles have several active metal sites available for rapid electron transfer and reactive oxygen species generation, and the porous UiO-66 structure facilitates dispersion, prevents agglomeration, and allows efficient mass transport of dye molecules. The synergetic interaction between the UiO-66 and MM matrix results in an efficient catalytic system with satisfactory mineralization efficiency. In general, the TOC analysis confirms that the MM@UiO-66 catalyst is not only capable of efficient dye degradation but also deep mineralization, which is of the greatest significance for actual wastewater treatment processes.

#### 3.2.4. Kinetics of MB photodegradation

Fig. S2 indicates photocatalytic decomposition of MB under optimal reaction conditions in a photoreactor. The time-resolved profiles indicate the proposed degradation pathway of MB at successive irradiation time intervals. Kinetic analysis was carried out to determine the apparent reaction rate constants and thus assess the material's photocatalytic activity. To obtain precise outcomes, the MM@UiO-66 photocatalyst and sample was used to estimate photocatalytic activity for different time periods. The MB degradation data were analyzed according to the pseudo–first-order kinetic model (eqn (3)):^31^

2 ln(*C*/*C*_0_) = −*Kt*

where *C*_0_ is the initial MB concentration, *C* is the concentration at time *t* (minutes), and *K* is the pseudo-first-order rate constant.

In the presence of MM@UiO-66 under irradiation, the MB absorbance band at 664 nm decreased continuously with time, indicating ongoing degradation. The rate constant of photocatalysis was calculated from the pseudo-first-order model, and *R*^2^ was shown in Table S1. The kinetics of the experiment showed pseudo-first order rate constants (*k*_1_) from 0.033 to 0.0432 min^−1^ for the MB concentrations ranging from 5–30 mg L^−1^, and there was a very good correlation between the data and the pseudo-first order model (*R*^2^ = 0.964–0.995) (see Table S1).

### 3.3. Photocatalytic degradation mechanism

**Fig. 10:**
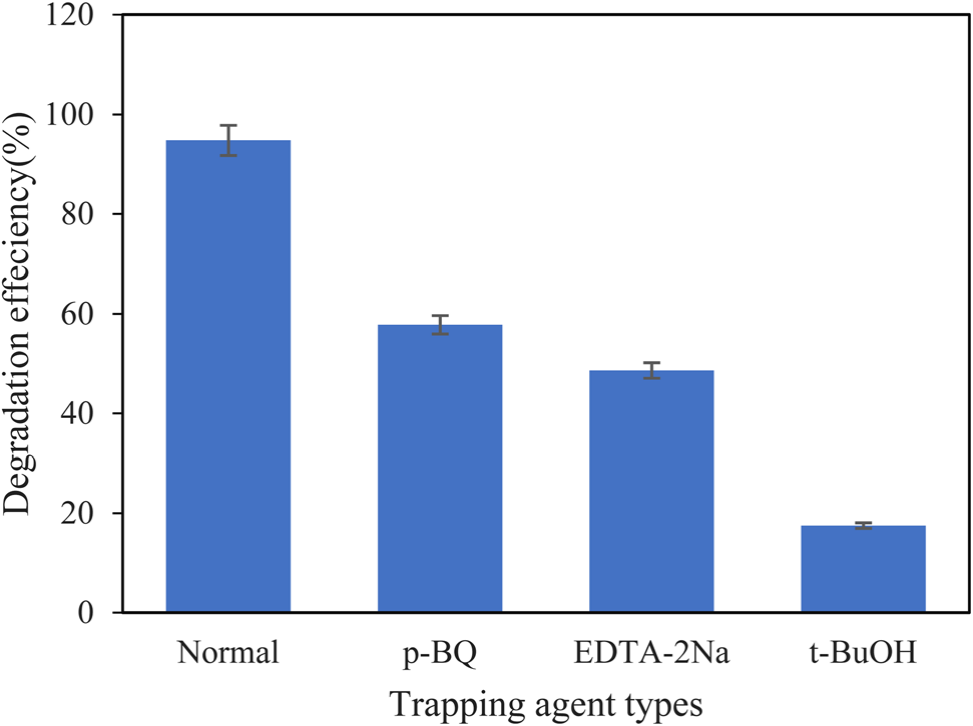
The effect of various scavengers on the photodegradation of MB.

The photocatalytic reaction mechanism and roles of various reactive oxygen species have been intensively investigated. The effect of various active species was investigated by scavenger experiments by introducing *p*-benzoquinone (*p*-BQ), *tert*-butyl alcohol (*t*-BuOH), and disodium ethylenediamine tetraacetate (EDTA-2Na) to the reaction solution; these are O_2_˙^−^, ˙OH, and h^+^ scavengers, respectively. The results of these extreme trapping experiments with MM@UiO-66 photocatalyst under visible light irradiation are shown in Fig. 10. It was found that the addition of *t*-BuOH distinctly impacted the rate of dye photooxidation, supporting the crucial role of ˙OH radicals during degradation. The photodegradation of MB was significantly inhibited when *t*-BuOH was added, further establishing the role of ˙OH. Similarly, EDTA-2Na decreased the overall photocatalytic efficiency of the suspension. These findings suggest that h^+^ and ˙OH radicals are the primary oxidative species, and O_2_˙^−^ radicals play a secondary role in the photocatalytic degradation of dye. Both ˙OH radicals and h^+^ are because they degrade MB through complementary mechanisms: h^+^ oxidizes directly electron-transferring surface-adsorbed MB molecules, whereas ˙OH radicals are diffusive species that permeate into the solution to decompose dissolved MB. The scavenger experiments also confirmed that quenching either of the species significantly inhibits degradation, meaning that neither is enough by itself—both must act simultaneously for the high efficiency of degradation observed. This dual mechanism is facilitated by the MM@UiO-66 composite, in which charge separation is facilitated by the multimetallic nanoparticles for maintaining h^+^ availability, and the porous UiO-66 structure provides extensive hydroxyl groups for ˙OH generation and MB enrichment near active sites.

Based on the scavenger tests, a likely charge transfer process for the MM@UiO-66 photocatalyst in visible-light-initiated MB degradation can be summarized as follows:

Electron–hole pairs:

3 MM@UiO-66 + *hν* → MM@UiO-66 + e^−^ + h^+^

4 MM@UiO-66 (e^−^)+ O_2_ → MM@UiO-66 + ˙O_2_^−^

5 MM@UiO-66(h^+^) + H_2_O → MM@UiO-66 + ˙OH + H^+^

6 ˙O_2_^−^ + H_2_O + e^−^ → ˙OH + OH^−^

7 ˙OH/˙O_2_^−^/h^+^ + MB → CO_2_ + H_2_O + other degradation compounds

**Scheme 2:**
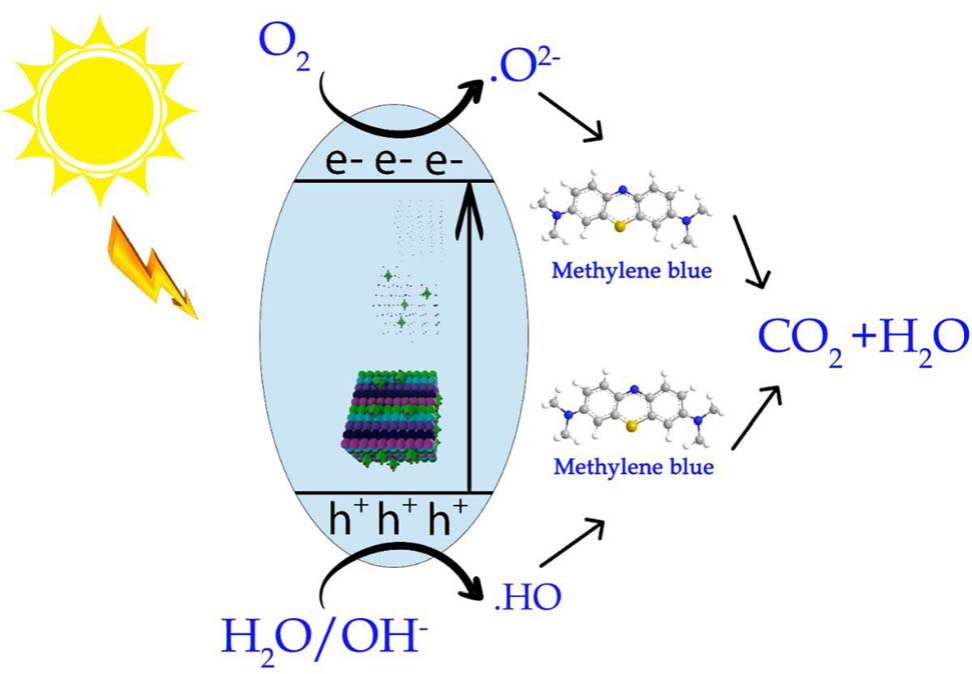
A possible mechanism for the degradation of MB on MM@UiO-66 nanocomposite.

Upon visible-light irradiation, charge carriers (electrons and holes) are generated within the MM@UiO-66 composite (eqn (3)). The photoexcited electrons are transferred to adsorbed oxygen on the surface of the catalyst to generate superoxide anion radicals (O_2_˙^−^) (eqn (4)). At the same time, the holes oxidize adsorbed water molecules, generating hydroxyl radicals (˙OH) and H^+^ ions (eqn (5)). The O_2_˙^−^ radicals may continue to react with water and electrons to produce more ˙OH radicals (eqn (6)). These active moieties (˙OH, O_2_˙^−^, and h^+^) catalyze MB's oxidative degradation to CO_2_, H_2_O, and other mineralized products (eqn (7)). Scavenger tests confirmed that ˙OH and h^+^ are the primary oxidative species, while O_2_˙^−^ is minor, confirming the overall photocatalytic process (Scheme 2).

### 3.4. UV-vis spectra and intermediate compounds

**Fig. 11:**
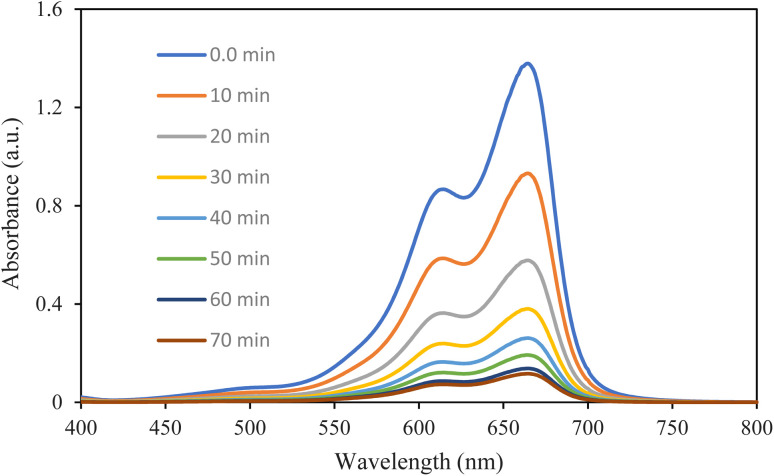
UV-vis spectrum of MB at different time intervals.

The photocatalytic degradation of methylene blue was monitored using UV-vis spectrophotometry with 70 minutes of irradiation using visible light (Fig. 11). The absorption spectra show a characteristic absorption peak of MB at around 664 nm, which is attributed to the n → π* electronic transition of the chromophoric conjugated structure of the dye molecule. As a function of the increase in irradiation time with visible light from 0 to 70 minutes, a progressive decline in the intensity of the 664 nm absorption was observed, indicating the gradual degradation of the MB dye. The maximum of absorption decreased from approximately 1.35 a.u. at 0 min to values approaching the baseline at 70 min, which was good photocatalytic activity of the (Mn, Co, Ni, Cr, Fe) MM@UiO-66 composite.

The degradation rate of the methylene blue (MB) dye was studied by UV-vis spectroscopy analysis within 70 min in the presence of visible light. It can be observed from the graphs that there is a distinctive peak of MB dye at a wavelength of ∼664 nm, which is due to the n → π* electronic transition of the chromophoric molecular structure of MB. In addition, with the increase in irradiation time from 0 to 70 min, there was a gradual reduction in the 664 nm peak of MB dye. The maximum absorbance reduced gradually from around 1.35 a.u. at 0 min to reach near the baseline value after 70 min irradiation. Therefore, this proves that there is good photocatalytic performance of the (Mn, Co, Ni, Cr, Fe) MM@UiO-66 material under visible light. Further, the scavenger's experiment was performed to determine the dominant reactive species in the photodegradation process. The findings show that both the ˙OH and h^+^ were significant reactive species for MB degradation, while O_2_˙^−^ was not important. Based on the results obtained above, a simplified scheme for the possible pathway of MB dye degradation is shown in Fig. S3. It is worth noting that the intermediates and products shown in Fig. S3 are purely hypothetical and only serve to illustrate the likely process of degradation conceptually.

Furthermore, to further validate the beneficial role of microwave-assisted irradiation, a control experiment was carried out by conventional heating (130 °C, 24 h) without microwave assistance. The conventionally synthesized sample showed lower photocatalytic efficiency toward methylene blue degradation, achieving 90.3% degradation, compared with 95.1% degradation for the microwave-assisted MM@UiO-66 composite under identical photocatalytic conditions. This result further confirms the practical advantage of microwave-assisted synthesis in obtaining a more active photocatalytic material within a significantly shorter synthesis time.

To evaluate the efficacy of the existing protocol, its efficiency was compared with previously reported methods. As listed in Table 1, MM@UiO-66 photocatalyst achieved 95.1% photodegradation of methylene blue under 70.0 min at as low as 20 mg L^−1^ compared to other processes listed in Table 1.

**Table 1:** Comparison of the degradation of MB in the literature report with the present work

Catalyst	Additive	Concentration (mg L^−1^)	Time (min)	Light source	Degradation (%)	Ref.
TiO_2_/FeS_2_	—	25	180	White lamps	97.0	32
MTCPP/TiO_2_	—	10	40	LED	93.0	33
ZnO-CuPc	0.1 mol L^−1^ NaOH	15	360	Philips	95.0	34
Cu–Zn-ferrite nanoparticles	—	10	35	UV-lamp	94	35
MM@UiO-66	—	10	70	LED	95.1	This work

The efficiency of the MM@UiO-66 composite for photocatalytic degradation of MB is presented in Table 1 and compared to other previously reported systems for MB photodegradation in terms of their efficiency. While the total degradation percentage of MM@UiO-66 (95.1%) is similar to the highest values recorded for other catalysts, closer analysis shows that the system under consideration possesses several practical benefits. First, contrary to the ZnO-CuPc and Cu–Zn ferrite composites, which need an alkali or ultraviolet irradiation, respectively, the current system does not require any additives and works effectively in the presence of visible LED light, which demonstrates its environmental friendliness. Moreover, while having a somewhat higher removal efficiency than the TiO_2_/FeS_2_ system, the latter needs significantly larger irradiation time (180 min). At the same time, despite the fact that the MTCPP/TiO_2_ composite demonstrates faster degradation, the MM@UiO-66 composite provides higher removal efficiency and sustainability, as seen from its reusability. Overall, beyond comparable degradation percentages, the combination of additive-free operation, visible-light activity, moderate reaction time, and catalyst stability positions MM@UiO-66 as a practically competitive and sustainable photocatalytic system.

### 3.5. Effect of water matrices

**Fig. 12:**
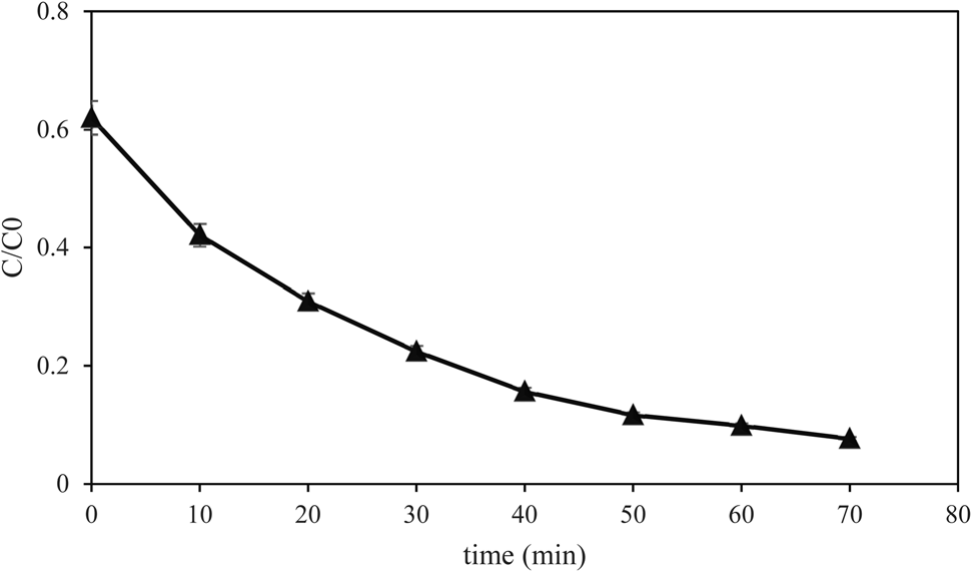
The effect of water matrix on the degradation of MB. Experimental condition: 20.0 mg, nanocomposite mass; 10.0 mg L^−1^, concentration of MB.

Natural water matrices are comprised of various constituents such as suspended particles, microorganisms, and dissolved organic matter that can compete with target pollutants for catalytic sites or diminish photocatalytic efficiency through light absorption. To assess the impact of real water matrices on MB degradation, experiments were conducted using municipal wastewater as a representative water source. Fig. 12 shows the degradation profiles of MB by 10.0 mg of the MM@UiO-66 composite in this matrix, indicating that the presence of naturally occurring matrix components does not significantly suppress the degradation efficiency, demonstrating the robustness of the photocatalytic system under realistic environmental conditions.

## 4. Conclusion

This study successfully synthesized for the first time a novel multimetallic nanoparticle supported by UiO-66 metal–organic framework (MM@UiO-66) nanocomposite by microwave-assisted one-pot synthesis with efficient photocatalytic degradation of methylene blue. The MM@UiO-66 composite exhibited outstanding photocatalytic activity, achieving 95.1% MB degradation under low-power LED illumination. Mechanistic exploration revealed hydroxyl radicals and photogenerated holes as the predominant oxidative species for dye degradation. The catalyst displayed higher reusability and stability with 90.8% degradation efficiency over six successive cycles. Metal–organic frameworks and MM are an emerging method for the design of future photocatalytic materials of high environmental remediation efficiency. The established synergistic advantages between MM multi-metal active sites and porous UiO-66 framework structures open new paths for designing efficient systems for wastewater treatment. The low-light illumination power-saving operation addresses critical sustainability challenges in industrial water treatment processes.

## Author contributions

Mostafa Khajeh: conceptualization; supervision; funding acquisition; project administration; methodology; validation, writing – original draft; writing – review & editing. Mansour Ghaffari-Moghaddam: conceptualization; methodology; writing – original draft; writing – review & editing. Afsaneh Barkhordar: data curation; investigation.

## Conflicts of interest

The authors state that no conflicts of interest exist in relation to this study.

## Supplementary Material

RA-016-D6RA03021A-s001

## Data Availability

Data supporting the findings of this study can be obtained from the corresponding author upon reasonable request. Supplementary information (SI): Fig. S1: The TOC concentration in the sample solutions for MB. Fig. S2: Linear regression fitting with a first-order model at different concentrations for MB. Table S1: The theoretical factors for fitting degradation data with First-order kinetic models by various concentrations of MB. Fig. S3: Degradation pathway of MB. See DOI: https://doi.org/10.1039/d6ra03021a.
