# Supplementary material for: Microwave-assisted one-pot synthesis of CrMnFeCoNi multimetallic nanoparticle-loaded UiO-66 for visible-light photocatalytic degradation of methylene blue
Source: RSC Adv. 2026 May 18;16(28):26143–54. doi: 10.1039/d6ra03021a (PMC13181363; doi:10.1039/d6ra03021a)
Supplement: RA-016-D6RA03021A-s001 [file RA-016-D6RA03021A-s001.pdf]

Supplementary Information (SI) for RSC Advances.
This journal is © The Royal Society of Chemistry 2026

# Microwave-Assisted One-Pot Synthesis of CrMnFeCoNi Multimetallic Nanoparticle-Loaded UiO-66 for Visible-Light Photocatalytic Degradation of Methylene Blue

Mostafa Khajeh[a,b] *, Mansour Ghaffari-Moghaddam[a,b], Afsaneh Barkhordar[a]

[a] Department of Chemistry, Faculty of Science, University of Zabol, Zabol, Iran

[b]Advanced Materials & Manufacturing Laboratory, University of Zabol, Zabol, Iran

* Corresponding authors.

*E-mail addresses:* m_khajeh@uoz.ac.ir (M. Khajeh)

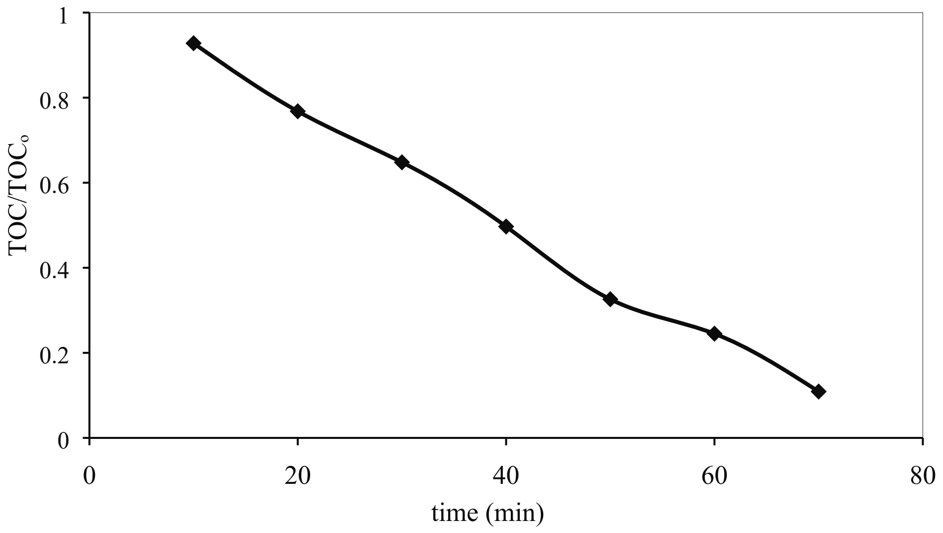


**Fig. S1** The TOC concentration in the sample solutions for MB

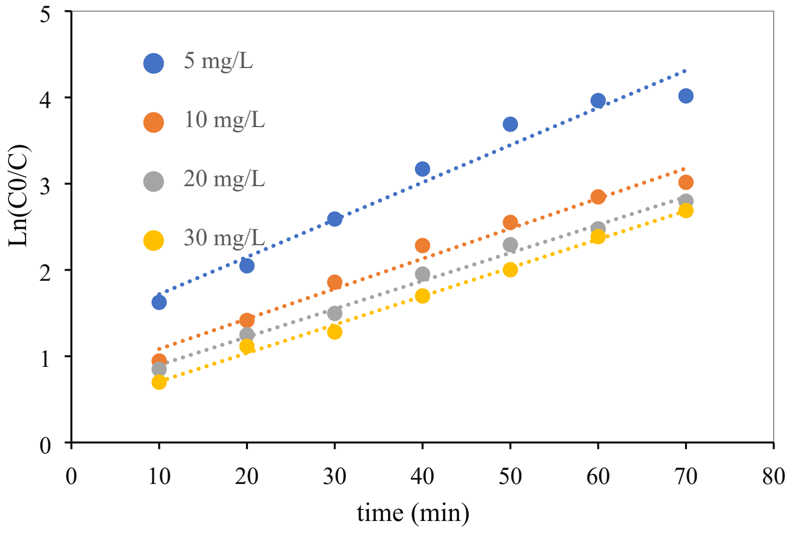


**Figure S2.** Linear regression fitting with a first-order model at different concentrations for MB

**Table S1.** The theoretical factors for fitting degradation data with First-order kinetic models by various concentrations of MB

| Concentration of MB (*mg L$^{-1}$*) | First order | |
|---|---|---|
| | $R^2$ | $K_1$ |
| 5 | 0.964 | 0.0432 |
| 10 | 0.977 | 0.0349 |
| 20 | 0.9914 | 0.0325 |
| 30 | 0.995 | 0.033 |

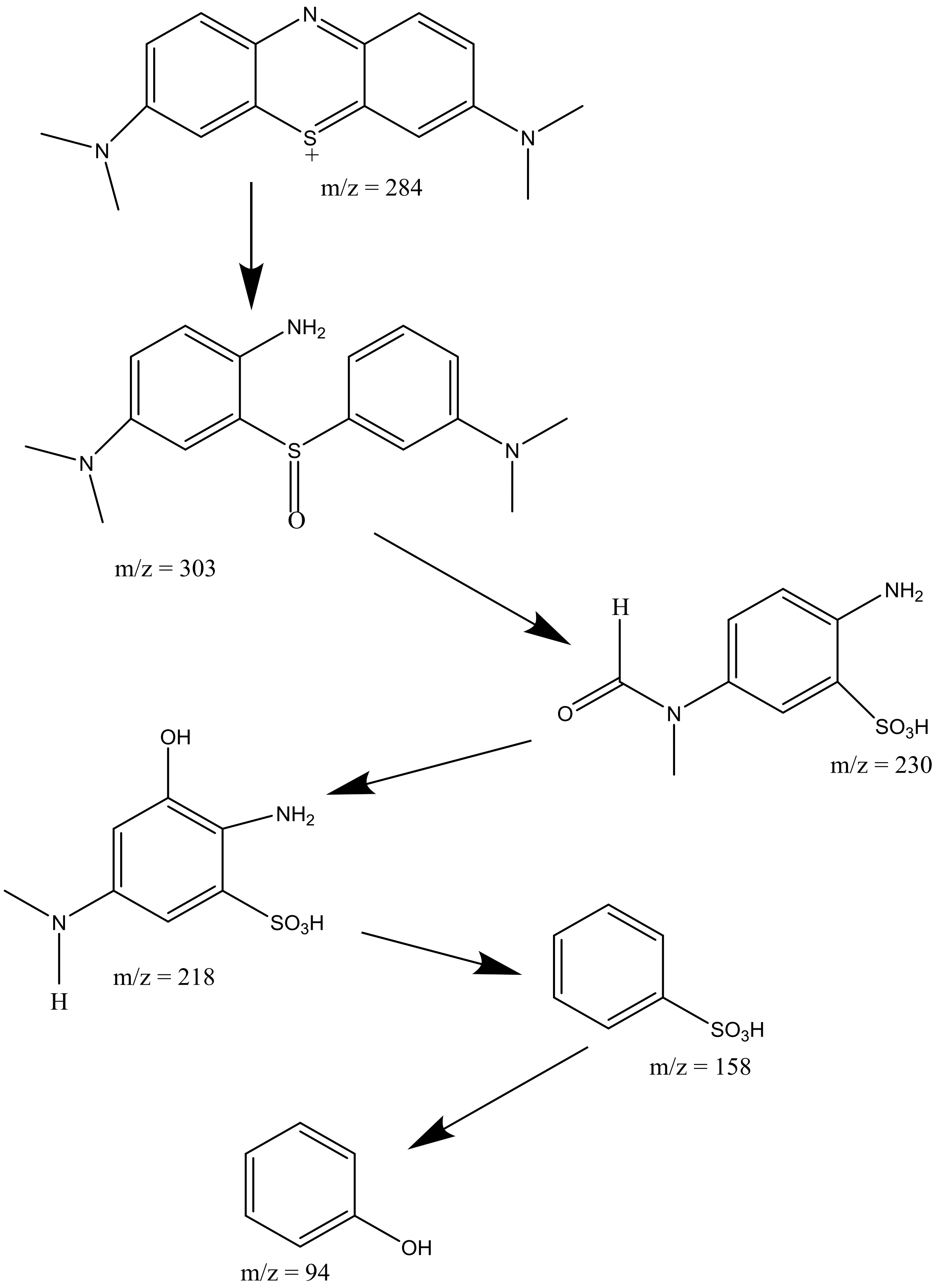


**Fig. S3** Degradation pathway of MB
